# Intelligent Fault Diagnosis of Industrial Bearings Using Transfer Learning and CNNs Pre-Trained for Audio Classification

**DOI:** 10.3390/s23010211

**Published:** 2022-12-25

**Authors:** Luigi Gianpio Di Maggio

**Affiliations:** Dipartimento di Ingegneria Meccanica e Aerospaziale (DIMEAS), Politecnico di Torino, Corso Duca Degli Abruzzi 24, 10129 Torino, Italy; luigi.dimaggio@polito.it

**Keywords:** intelligent fault diagnosis, deep learning, transfer learning, rolling bearings, bearing test rig, condition monitoring

## Abstract

The training of Artificial Intelligence algorithms for machine diagnosis often requires a huge amount of data, which is scarcely available in industry. This work shows that convolutional networks pre-trained for audio classification already contain knowledge for classifying bearing vibrations, since both tasks share the need to extract features from spectrograms. Knowledge transfer is realized through transfer learning to identify localized defects in rolling element bearings. This technique provides a tool to transfer the knowledge embedded in neural networks pre-trained for fulfilling similar tasks to diagnostic scenarios, significantly limiting the amount of data needed for fine-tuning. The VGGish model was fine-tuned for the specific diagnostic task by handling vibration samples. Data were extracted from the test bench for medium-size bearings specially set up in the mechanical engineering laboratories of the Politecnico di Torino. The experiment involved three damage classes. Results show that the model pre-trained using sound spectrograms can be successfully employed for classifying the bearing state through vibration spectrograms. The effectiveness of the model is assessed through comparisons with the existing literature.

## 1. Introduction

The monitoring of rotating systems through bearing sensoring is part of the implementation of predictive maintenance strategies. The deployment of such approaches is motivated by the resulting benefits for industrial rotors in terms of cost reduction and increased production [1]. A primary concern of predictive maintenance and condition monitoring is the fault diagnosis of bearings, this is for two main reasons. First, durability assessments of rolling bearings are affected by significant uncertainties [2], given the complex interaction between a variety of parts. Additionally, it is well established that bearings are key nodes for retrieving information on the whole mechanical system [3]. In this context, the analysis of vibration signals represents one of the most informative tools for the assessment of machine conditions [4].

The past thirty years have seen increasingly rapid advances in this field thanks to the development of numerous signal processing techniques for fault identification. For instance, the literature on envelope analysis has been considerably developed [2,4,5,6,7,8,9,10,11], which has shown its effectiveness in benchmark cases [12] and it is being implemented in industry for condition monitoring purposes. The outcomes of this kind of signal processing tool have the benefit of being highly interpretable, since the models’ assumptions are sharply identifiable. On the other hand, the outcomes may be user dependent. The extraction of diagnostic information from vibration signals is often affected by the assumptions of the identification models and by the user’s experience. For instance, choosing an optimal demodulation band [13,14,15,16,17] naturally implies an inherent arbitrariness.

Conversely, data-driven models rely on Artificial Intelligence (AI) algorithms in order to automatically learn fault detection abilities from training data [18,19,20,21]. Although these structures can fulfil highly complex tasks, it is fairly challenging to figure out the rationale behind models’ decisions [22,23]. The choice and the extraction of fault features can either be manual, as in the case of the Support Vector Machine (SVM) algorithm [24,25,26,27], or automated, as in the case of the application of deep learning to several disciplines [28,29,30]. Manual feature extraction is performed prior to the training by selecting features. Most of the literature concerning deep learning involves Convolutional Neural Networks (CNNs). For instance, Guo et al. trained a CNN using wavelet time-frequency images extracted from vibration signals [31], Wen et al. [32] developed a signal-to-image conversion method for training CNNs and Islam et al. [33] fed a CNN by employing acoustic emission (AE) data.

One of the major drawbacks of neural networks is the amount of data needed for training, because the number of parameters to be trained is much higher than that of machine learning algorithms. Some research areas benefit from million-sample datasets to accomplish challenging tasks, as in the case of ImageNet [34,35] for image recognition and Audio Set [36,37] for audio classification. At present, the datasets involving machine vibrations [12,38,39,40] do not contain such a huge amount of samples, and industrial environments rarely have a wide range of fault data. Additionally, vibration signals are tightly connected to a specific machine and operating conditions. Therefore, the employment of large and high potential networks could produce diagnosis models that overfit training data, losing the ability to generalize diagnostic patterns to real test conditions. Additionally, the existing literature emphasizes issues in some benchmark datasets, such as the CWRU [12,41], which may be moreover not suitable for investigating industrial-size bearings. 

Recent evidence suggests the applicability of transfer learning (TL) [20,42,43] to tackle these issues in the field of machine fault diagnosis. TL aims to reduce data collection by transferring the classification knowledge of pre-trained models to new domains or new tasks. Zhang et al. [44] and Cao et al. [45] showed that knowledge transfer can be realized within the same machine whenever the user wishes to apply a trained model to new operating conditions. Compound faults were analyzed by Hasan et al. [46], whereas Wang et al. investigated RUL estimations [47]. Instead, Guo et al. [48] transferred a convolutional diagnosis model across different machines, whereas Chao et al. performed online domain adaptation [49]. Similarly, improved transfer learning with hybrid feature extraction was proposed by Yang et al. [50]. Han et al. [51] employed joint distribution adaptation. The Generative Adversarial Networks (GANs) approach was investigated by Li et al. [52], Shao et al. [53] and Wang et al. [54]. Nonetheless, recent works have showed that fault diagnosis tasks can be fulfilled on benchmark datasets by employing AI frameworks originally designed for completely different tasks such as image recognition [55] and audio classification [56]. However, to the best of the author’s knowledge, few studies have investigated the performances of the latter algorithms on industrial cases characterized by medium-sized bearings.

Firstly, the purpose of this investigation is to analyze a new dataset for bearing fault detection, specifically conceived for medium-sized bearings of industrial interest. Indeed, the well-known CWRU dataset presents several issues which were discussed by Smith and Randall in 2015 [12] and by Hendriks et al. in 2022 [57]. The findings of [12,57] suggest that CWRU data may be not representative of bearing faults in general and, even more so, of the industrial case analyzed in this paper. Additionally, this study is motivated by the fact that although CNNs and TL were deeply analyzed in the literature concerning bearing fault diagnosis, the capabilities of CNNs pre-trained for audio classification have been investigated very little. Indeed, the literature has mostly focused on transferring knowledge from CNNs pre-trained for image recognition [55]. According to the author of this work, CNNs for audio classification deserve to be explored further since, unlike image recognition networks, these frameworks already contain a highly specific knowledge for extracting spectrogram features. 

This paper discusses the application of a transfer learning methodology to the test rig available at Politecnico di Torino [58], which was designed to accommodate medium-sized bearings of industrial interest. To the best of the author’s knowledge, this is the first work including experiments conducted on medium-size industrial bearings with localized faults. Additionally, this paper aims to explore the fault diagnosis capabilities of CNNs pre-trained for audio classification. Namely, the VGGish convolutional network [37,59,60] is employed to perform bearing fault diagnosis. The VGGish network was originally trained for large-scale audio classification by using millions of audio samples extracted from YouTube® videos [37]. In this work, the pre-trained model is fine-tuned by a few thousands vibration records retrieved under different working conditions of the machine. Such a knowledge transfer is inspired by the idea that the search of fault distinctive features in vibration spectrograms is conceptually similar to the identification of sound spectrograms [56]. The results corroborate this hypothesis and show that the feature extraction capabilities of the pre-trained VGGish network can be effectively transferred to fault diagnosis scenarios. Thanks to the use of TL, a large-scale and high-potential classification model can be reused for the purpose of machine diagnosis by fine-tuning with a very small dataset. Furthermore, it is shown that the pre-trained VGGish model outperforms the VGGish framework trained from scratch in the presence of a thousand-sample set. Additionally, it is found that, for the case under analysis, the VGGish performs better than models pre-trained for image recognition.

The overall structure of this paper takes the form of five sections. The introductory paragraph presents the topic of intelligent fault diagnosis of industrial bearings and provides the motivations which motivated the author to perform this investigation with respect to the existing literature. The second section gives an insight into the AI methodologies involved in this study, and CNNs, transfer learning and the VGGish model are presented. A description of the test rig for industrial bearings and the vibration dataset is provided in the third section, whereas the fourth section includes results, discussion and implications. Finally, the fifth section provides the concluding remarks.

## 2. Transfer Learning for Bearing Fault Diagnosis

This section provides a short summary of the main AI devices involved in this study. CNNs, transfer learning and the VGGish audio feature extractor are introduced.

### 2.1. Convolutional Neural Networks (CNNs)

**Figure 1:**
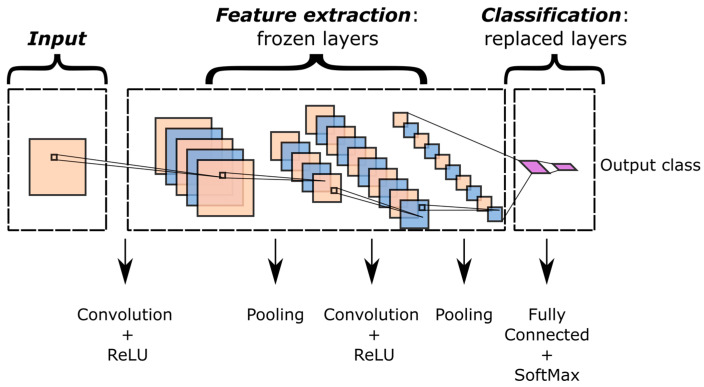
Typical transfer learning framework in CNNs.

The typical structure of a CNN (Figure 1) includes a sequence of layers in which several algebraic operations take place. This claim is valid for the vast majority of deep learning approaches, but CNNs are differentiated by their ability to handle multidimensional data. That is one of the reasons why the introduction of CNNs [34,61] completely transformed image-based AI. A wide range of research areas thereafter took advantage of these structures. Indeed, as previously described, sound spectrograms were employed to train CNNs for audio classification [37]. The convolution operation mainly consists of applying filter kernels to the input data, whereas pooling layers carry out data downsampling. Finally, fully connected layers flatten multidimensional data [18,20,55,56] in one-dimensional vectors. For classification tasks, the last fully connected layer returns the output class. Convolutional and fully connected layers also implement nonlinear effects by means of activation functions. The Rectified Linear Unit (ReLU) is one of the possible activation functions for introducing nonlinearities in the output of convolutional layers [56].

The training process aims to optimize a specific loss function, which can be interpreted as a measure of the distance between the predictions of the model and the ground truth. For instance, the cross-entropy of Equation (1) is the typical loss function employed for classification tasks with mutually exclusive classes,

(1) $$Loss=-\frac{1}{M}\overset{M}{\underset{m=1}{\sum }}\overset{N}{\underset{n=1}{\sum }}y_{mn}\ln{\hat{y}}_{mn}$$

where:

- M is the number of observations;
- N is the number of classes;
- ${\hat{y}}_{mn}$ is the network output for the m-th observation and the n-th class;
- $y_{mn}$ is the ground truth for the m-th observation and the n-th class.

At the end of training stage, the weights of the network filters are optimized for the specific task and contain the knowledge related to the latter. In particular, the stacked convolutional layers learn hierarchical representations of the input data. Convolutional layers are mainly devoted to the feature extraction. For deep learning models, the extraction process is automated and does not require manual feature selection. Moreover, deeper layers correspond to more abstract features. In other words, the convolutional layers learn to extract discriminating features of the input data during training. The extracted feature maps are condensed in the fully connected layers which terminate in the network output for the classification.

### 2.2. Transfer Learning

Transfer learning covers a wide range of techniques aimed at reusing the knowledge already contained in AI models. A complete exploration of all the TL methodologies is beyond the scope of this study; a comprehensive insight is given by the works of Pan and Yang [42] and Lei et al. [20]. Parameter-based TL is considered for the purpose of this investigation. Namely, it is assumed that the knowledge transfer can be carried out by reusing the parameters of a pre-trained model. In the case of CNNs, the parameters are represented by the network weights, which enclose the knowledge. Thanks to the data from the source domain, the pre-trained network acquires the feature extraction capabilities for accomplishing the specific source task. The knowledge is thus transferred to the target domain of interest to fulfil a target task.

Figure 1 shows a typical transfer learning framework for CNNs. Some or all of the feature extraction layers are frozen, whereas the last layers are replaced with new ones. The weights of the latter are optimized by fine-tuning the model in the target domain. One of the most fascinating aspects of this technique is related to the amount of training data. Considering that the actual training involves few layers, the amount of training data is extremely low with respect to training from scratch. However, the potential of extracting complex features is preserved in the frozen layers.

**Figure 2:**
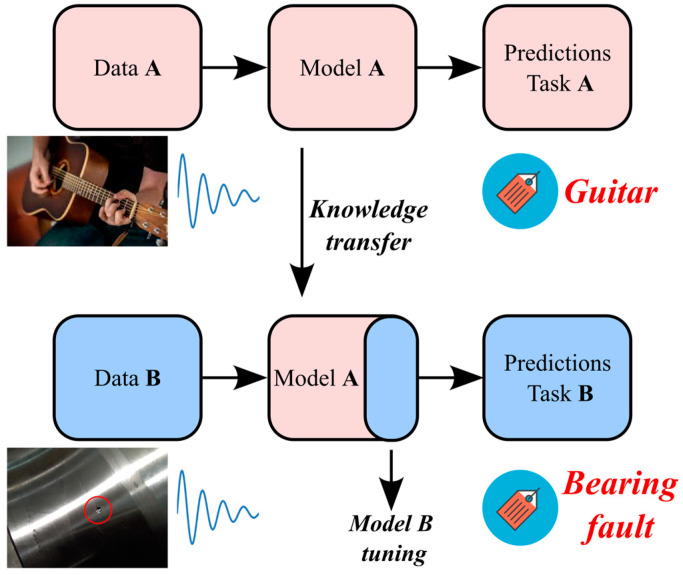
Knowledge transfer from an audio feature extractor to bearing health monitoring.

This study investigates the case of knowledge transfer from an audio feature extractor to the assessment of bearing health state. The methodology is outlined in Figure 2. The model A is pre-trained for audio recognition. For instance, the label “Guitar” is assigned to guitar sounds. The ability of extracting spectrogram features is transferred to the domain of vibration signals by reusing part of the model A. Then, the model B is fine-tuned by employing a reduced amount of target data. As an example, the target task could be the assignment of the label “Bearing fault” to the vibration signal.

### 2.3. VGGish Network for Bearing Health Monitoring

An audio feature extractor is a CNN designed to unpack the most distinctive features detectable in an audio spectrogram. These features are condensed in a low-dimensional space, where a classifier can operate more conveniently to discern classes. This process is also known as feature embedding. The classifier can also be constituted of a series of fully connected layers attached to the end of the feature extractor. The author chose to transfer knowledge from an audio CNN because those networks can already identify spectrogram features, wherever the signal originates. However, the literature shows examples of knowledge transfer from image classification networks [55] to benchmark vibration datasets.

**Figure 3:**
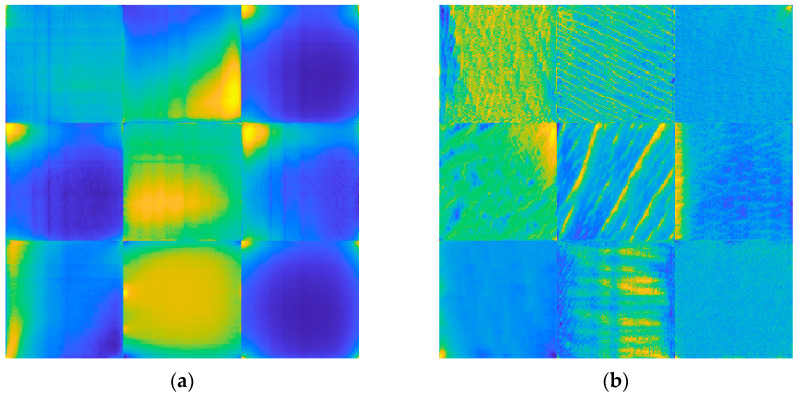
VGGish layers: (**a**) example of features learned in the layer Conv 2; (**b**) example of features learned in the layer Conv 3_1.

The VGGish architecture [37] summarized in Table 1 contains 62 million weights. The model was originally trained by Hershey et al. [37] in 2017 by using 70 million YouTube® clips, for a total amount of 5.24 million hours and 30,871 audio labels. The network input is constituted of a 96×64 mel spectrogram [62,63], which is a time-frequency transformation typically applied to audio signals. The pre-trained framework can be used in two ways. First, it can act as a feature extractor to embed audio in the 128 feature vector that feeds a classification model. Alternatively, the architecture can be part of a larger model that needs fine-tuning. Figure 3a and Figure 3b show examples of low-level and medium-level features, respectively, learned by the pre-trained VGGish. It is noted that more complex spectrogram features correspond to deeper layers.

Some preprocessing is needed to feed the VGGish architecture:

- Signals are resampled at 16 kHz and normalized in the range [−1, 1];
- Each frame is converted in a log-mel spectrogram [62,63] of 64 frequency bins covering the range 125–7500 Hz by applying 25 ms windows every 10 ms;
- Mel spectrograms are framed into samples of 0.96 s, which correspond to 96 frames of 10 ms.

The preprocessing steps result in a 96×64 patch, in accordance with the input of the network. The use of the mel spectrogram [62,63] is quite common in audio processing. Indeed, the mel scale is perceptually relevant for human hearing, which is more sensitive at lower frequencies. In this study, the same preprocessing steps are applied to vibration signals in order to enhance the similarities between the source and the target domain. According to the author of this work, it is reasonable to assume that this circumstance fosters knowledge transferability.

TL was applied for identifying bearing health conditions. For this purpose, the last layer of the VGGish was replaced with a new one. Namely, the regression layer was replaced with a fully connected layer with three neurons for classifying three bearing health states. Next, a classification layer was added. Since the feature extraction layers remained unchanged, it can be stated that the original VGGish feature embedding fed the classification layer. Moreover, a dropout layer was added before the last fully connected layer. Dropout layers set weights to zero with a given probability in order to reduce the number of trainable parameters and avoid overfitting. In this case, the dropout probability was set to 50%. When the training was run, only the weights related to new layers were updated. The replacement of the only last layer and the implementation of dropout strategies showed to be the most effective approach for the analyzed case. Table 2 reports the set of hyperparameters adopted in this work.

## 3. Vibration Dataset for Industrial Bearings

The TL methodology was applied to the dataset generated by a test rig for industrial bearings available at Politecnico di Torino [58]. To the best of the author’s knowledge, the existing literature provides scant evidence of deep learning strategies applied to datasets covering medium-size bearings (360 mm outer diameter). Three health states were analyzed: normal condition, inner race damage and outer race damage. This section provides a description of the test rig, of the experimental activity and of the dataset construction.

### 3.1. Description of the Test Rig

**Figure 4:**
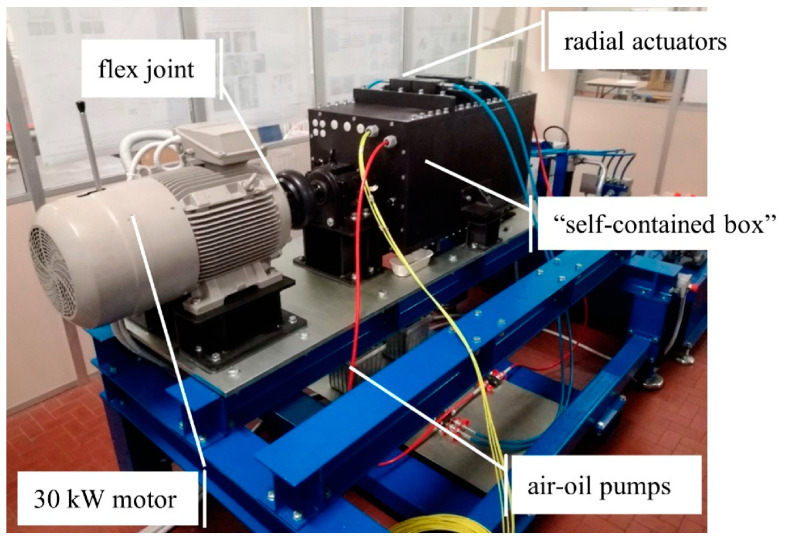
Test rig for industrial bearings [58].

The test rig presented in reference [58] (Figure 4) can house up to four bearings with outer diameters ranging from 280 mm to 420 mm. A full description of the test rig goes beyond the scope of this work, since a comprehensive outline of the design activity and equipment is already provided in [58]. A 30 kW three-phase induction motor is controlled by an inverter. The motor is connected to the shaft by means of a rubber joint. The shaft rotation is sustained by the two main bearings. The so-called “self-contained box” houses the test bearings, which can be loaded with up to 200 kN thanks to oil actuators. The two air-oil pumps control the radial and the axial actuators, respectively, by converting pneumatic pressure into oil pressure (up to 500 bar). Then, the radial and the axial loads are applied independently. The lubrication system consists of an external control unit that monitors the oil jet system. The ISO VG 150 oil is injected with a flow rate of 2.5 L/min and a pressure of 6 bar.

**Figure 5:**
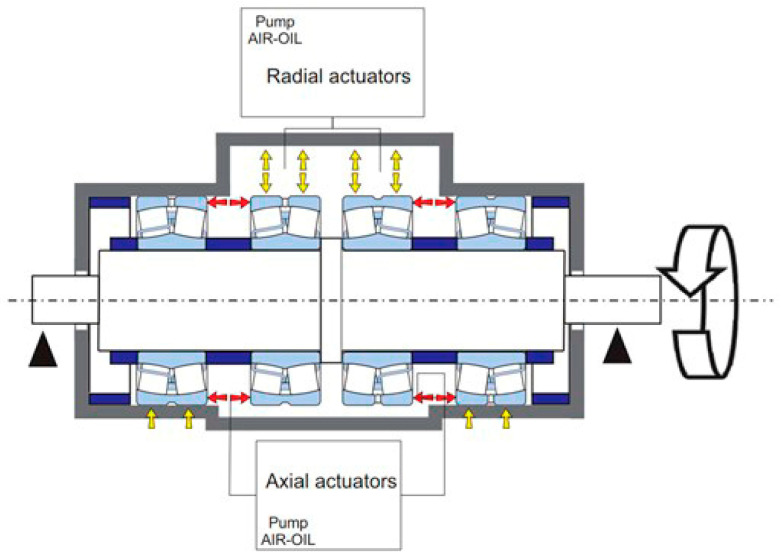
Scheme of the self-contained box [58].

The layout of the self-contained box (Figure 5) provides an advantage of balancing the loads of the actuators through the elastic deformation of the box. Thus, the test loads are internally accommodated and the load circuit is “self-contained”. Consequently, the main bearings do not have to fulfil stringent requirements in terms of strength and minimum size. The test bearings can be replaced by resorting to proper adapters. The purpose of the adapters is to comply with the size of the box regardless of the outer diameter of the bearings. 

Four SKF CMS 2200T sensors are fitted to the four adapters in order to measure acceleration and temperature. The main features of the vibration sensors are reported in Table 3. The condition monitoring framework includes a LMS Scadas III data acquisition system. The latter is interfaced with a laptop for signal acquisition and post-processing.

### 3.2. Experimental Activity and Dataset Construction

**Figure 6:**
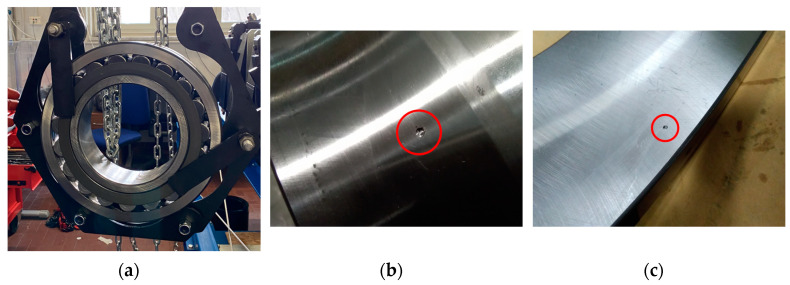
SKF 22,240 CCK/W33: (**a**) normal state bearing during dismounting; (**b**) inner race damage with 2 mm diameter and 0.5 mm depth; (**c**) outer race damage with 2 mm diameter and 0.5 mm depth.

This study takes into account three health states for the spherical roller bearing SKF 22,240 CCK/W33 (Figure 6a). The bearings have an inner diameter of 200 mm with a 1:12 tapered bore and an outer diameter of 360 mm. In addition to the normal state, inner race (IR) damage (Figure 6b) and outer race (OR) damage (Figure 6c) are considered. The faults have a diameter of 2 mm and a depth of 0.5 mm. The damages were mechanically machined on the race that is most loaded in the case of application of an axial load. In order to apply the damages, bearings were dismounted. Then, the faults were drilled on the race of interest by employing a solid carbide drill with a diameter of 2 mm. Although the produced faults are representative of localized defects in rolling bearings, the vibration data extracted cannot obviously represent the complete scenario of defects detectable in rolling bearings.

The experiment involved the analysis of four load cases at 10 different shaft speeds as reported in Table 4. Then, 40 signals were extracted for each health state totaling 120 signals. The vibration signals were acquired by means of the data acquisition system and sampled at 20,480 Hz. Each of the acquisitions lasted 30 s. Therefore, 1 hour of signal acquisition was taken into account.

The dataset was constructed by extracting non-overlapping chunks from the vibration signals (Table 5). The duration of the chunks was of 1.6 s. Therefore, 18 chunks were extracted for each signal. The resulting dataset consisted of 2160 samples equally balanced in the three classes: Normal, IR and OR. The data labelling for the supervised learning scheme was achieved as a natural consequence of the experiment. The amount of data are remarkably low for the use of large deep learning architectures. However, fault diagnosis can be performed thanks to TL.

The dataset was randomly split in order to test the applicability of the proposed method. Table 6 reports the information regarding the data split. A typical deep learning splitting strategy was applied: 80% of the data were used for fine-tuning the VGGish model, 10% of the data constituted the validation set, whereas the remaining 10% were used to test the method with new data. 

## 4. Results and Discussion

**Figure 7:**
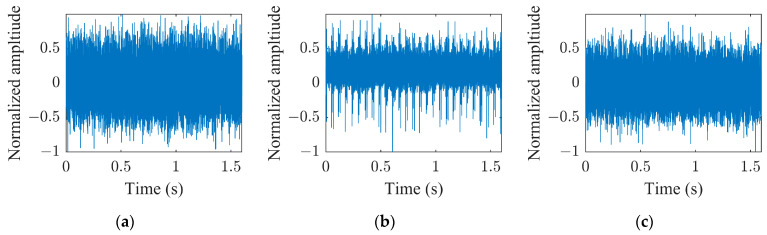
Vibration signal at 997 rpm and 124.8 kN radial load: (**a**) normal health state; (**b**) IR damage; (**c**) OR damage.

**Figure 8:**
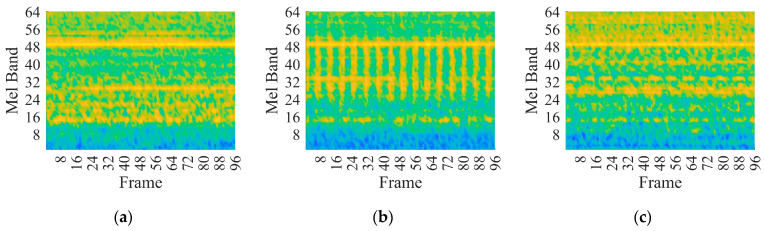
Mel spectrogram at 997 rpm and 124.8 kN radial load: (**a**) normal health state; (**b**) IR damage; (**c**) OR damage.

**Figure 9:**
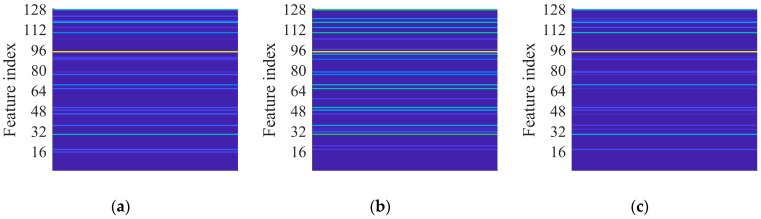
VGGish feature embedding at 997 rpm and 124.8 kN radial load: (**a**) normal health state; (**b**) IR damage; (**c**) OR damage.

This paper investigates the capabilities of CNNs pre-trained for audio classification to perform bearing fault diagnosis. It is argued that these networks are endowed with highly specific knowledge for extracting spectrogram features. For this purpose, the vibration dataset including damaged industrial medium-sized bearings was produced by means of proper experimental activity conducted on a specifically conceived test rig. A detailed description of the hardware is provided in reference [58]. As anticipated in Section 2.3, the VGGish convolutional architecture can act as a spectrogram feature extractor, as long as a proper preprocessing is carried out. Figure 7a, Figure 7b and Figure 7c show examples of normalized vibration signals for the normal state, IR and OR damages, respectively. Figure 8a–c shows the corresponding mel spectrograms obtained through the preprocessing. Finally, Figure 9a–c shows the corresponding 128-dimensional feature embedding output from the pre-trained VGGish feature extractor. Essentially, the information dissolved in the multifaceted mel spectrograms is translated and synthetized in a low-dimensional feature space via feature embedding. The classifier can discern classes by learning the differences that establish between feature embeddings. In this particular case, the feature embedding corresponds to a vector containing 128 elements.

**Figure 10:**
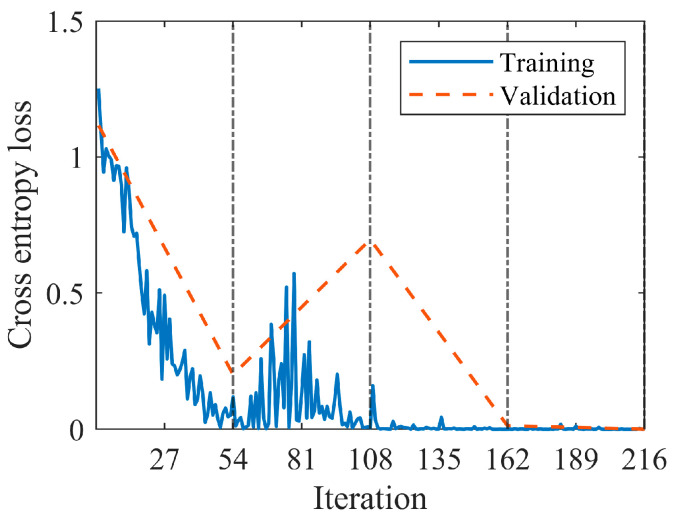
Loss functions.

**Figure 11:**
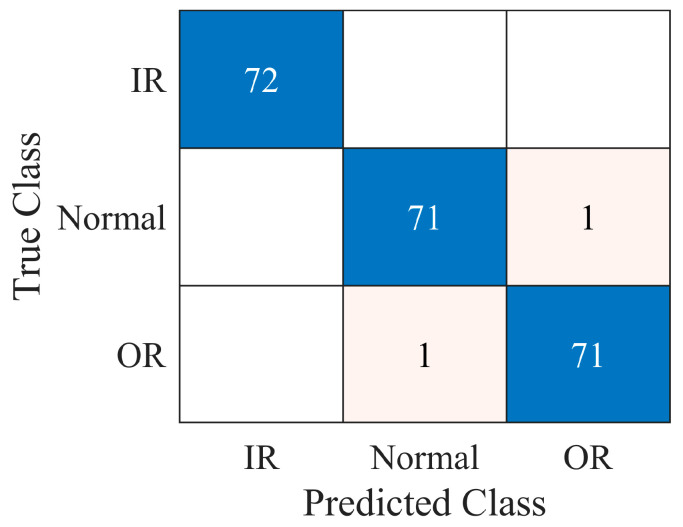
Test confusion matrix.

The model was fine-tuned using the hyperparameters reported in Table 2. The training time was 936 s on a standard laptop without GPU acceleration (Intel^®^ Core i7−10510U CPU @ 1.80 GHz). The model was implemented in the Matlab^®^ environment by means of machine learning, deep learning and audio toolbox libraries. It is worth noting that the original VGGish structure was trained on multiple GPUs for 184 hours [37]. Figure 10 shows the behavior of the loss functions during the training conducted according to the parameters in Table 2. In particular, the validation set served to monitor potential overfitting by analyzing the trend in the validation loss. The number of maximum epochs was set to four (216 iterations), since it was observed that the training process stabilized at this point and overfitting did not occur, though it was detectable during the first two epochs. The accuracies reported in Table 7 reveal the applicability of the diagnosis model to new test data. The complete confusion matrix resulting from the test data is shown in Figure 11. A single normal sample is predicted as OR damaged and a single OR sample is predicted as normal. Therefore, the classifier showed high precision and recall as reported in Table 8.

Furthermore, the proposed model was compared with the VGGish model trained from scratch, the YAMNet model [56] and the VGG16 model pre-trained on ImageNet [34,35] proposed by Shao et al. [55]. Table 7 shows the accuracies obtained for the different models, whereas Table 8 reports the precision and the recall for the different classes. The VGGish trained from scratch reaches poor diagnosis accuracies and consistent overfitting phenomena occur. This is due to the fact that the original VGGish architecture was trained on millions of samples. Therefore, the structure is inherently unsuitable for correctly learning hierarchical features over a few thousands of training samples. Given the availability of a limited amount of training data, network weights of millions are extremely prone to overfit the training set. For this reason, TL is the most effective strategy. The YAMNet model [56] showed promising accuracies and reduced training times, but some overfitting was detectable. Finally, the VGG16 model [55] was trained by employing wavelet time-frequency images. The training of the model under the conditions reported in [55] required GPUs and was computationally expensive. The resulting metrics show that the VGG16 framework pre-trained on ImageNet is not suitable for the analyzed case. According to the author of this work, this is due to the fact that several convolutional layers should be retrained in the model [55]. Consequently, more training data are required. On the other hand, few layers of the pre-trained VGGish and YAMNet need fine-tuning, since audio classification models are already capable of extracting distinctive spectrogram features. On the contrary, the knowledge contained in networks pre-trained on the ImageNet dataset cannot be considered highly specific for spectrogram recognition.

The encouraging results indicate that the TL methodology is a valuable approach for the fault diagnosis of bearings. Remarkably, the knowledge contained in a network pre-trained for sound recognition can be reused for condition monitoring tasks. Moreover, the amount of training data is considerably low with respect to the network trained from scratch. The original VGGish network was trained by using 70 million audio samples, whereas less than 2000 samples were needed for performing fault diagnosis. Therefore, deep learning frameworks endowed with high knowledge content could be exploited without the need for millions of data samples. This remarkable implication is determined by the fact that the features extracted from the pre-trained VGGish network are already capable of identifying typical spectrogram features. Then, only slight adjustments are needed to adapt the model to the classification of vibration spectrograms. The feature embedding in which the sound spectrograms are translated is therefore convenient for vibration spectrograms as well. 

However, this occurrence poses an issue in the interpretation of the diagnosis outcomes. Indeed, the 128 features which flow through the classifier have no clear physical interpretation. In this case, acoustically relevant features were able to classify vibrations. In contrast to traditional signal processing tools, where some parameters (e.g., kurtosis, crest factor and ball passing frequencies) have a physical meaning, the user does not know what the features actually represent for data-driven fault diagnosis, although they may perfectly work. Therefore, it is quite challenging to estimate the features variability with respect to the changes in the input signals. Additionally, the development of proper interpretability tools is of paramount importance for the correct visualization of domains alignment in transfer learning.

## 5. Conclusions

This work proposes a transfer learning methodology for fault diagnosis of industrial bearings. The VGGish architecture, originally pre-trained for sound classification on 70 million audio samples, is fine-tuned by using less than 2000 vibration samples. The experimental data related to the test set-up at the Politecnico di Torino and designed for the monitoring of industrial bearings are hereby presented. The experiment involved three health states ranging over ten speeds and four load cases for medium-size bearings. Vibration data were classified with 99.07% accuracy. The training time was 936 s. It is concluded that:

- Deep learning CNNs are promising approaches for industrial condition monitoring;
- The existing potentials included in large deep learning architectures can be exploited for bearing fault diagnosis using of small datasets, as long as transfer learning is applied;
- Transfer learning drastically reduces the computational demand by applying deep learning in fault diagnosis tasks;
- The acoustical features extracted from the VGGish network are also relevant for classifying bearing vibrations;
- CNNs pre-trained for sound classification are more efficient and accurate than models pre-trained for image recognition.

The main limitations include the challenge of interpreting the extracted features. Although this study exhibits promising results, further investigations are also needed to apply this concept in industry, where fault data are scarcely available and balanced classes are not applicable. The knowledge transfer to unseen working conditions or different machines should be investigated as well.

## Figures and Tables

**Table 1:** VGGish layers.

Layer	Type	Filter Size	Number of Channels	Activation Function
Input	Image Input	96×64×1	1	−
Conv 1	Convolution	3×3×1	64	ReLU
Pool 1	Max Pooling	2×2	−	−
Conv 2	Convolution	3×3×64	128	ReLU
Pool 2	Max Pooling	2×2	−	−
Conv 3_1	Convolution	3×3×128	256	ReLU
Conv 3_2	Convolution	3×3×256	256	ReLU
Pool 3	Max Pooling	2×2	−	−
Conv 4_1	Convolution	3×3×256	512	ReLU
Conv 4_2	Convolution	3×3×512	512	ReLU
Pool 4	Max Pooling	2×2	−	−
Fc 1_1	Fully Connected	4096	−	ReLU
Fc 1_2	Fully Connected	4096	−	ReLU
Fc 2	Fully Connected	128	−	ReLU
Output	Regression Output	−	−	−

**Table 2:** Hyperparameters for VGGish transfer learning.

Hyperparameter	Value
Optimizer	Adam [64]
L2 regularization	1 × 10^−6^
Mini batch size	32
Iterations per epoch	54
Initial learning rate	5 × 10^−4^
Learning rate drop period	2
Learning rate drop factor	0.5
Max epochs	4

**Table 3:** SKF CMS 2200T sensor specifications.

Sensitivity	100 mV/g
Sensitivity precision	±5% at 25 °C
Acceleration range	60 g peak
Amplitude linearity	1%
Resonance frequency, mounted, minimum	22 kHz
Frequency range	±5%: 1.0 to 5000 Hz
	±10%: 0.7 to 10,000Hz
	±3 dB: 0.5 to 12,000Hz

**Table 4:** Test conditions.

	Load Case 1	Load Case 2	Load Case 3	Load Case 4
Radial load (kN)	0	64	124.8	124.8
Axial load (kN)	0	0	0	49
Nominal speeds (rpm)	127, 227, 353, 457, 523, 607, 727, 877, 937, 997			

**Table 5:** Signal extraction.

Total acquisition duration (s)	30
Sampling frequency (Hz)	20,480
Chunk length (samples)	32,768
Chunk length (s)	1.6
Number of chunks per signal	18

**Table 6:** Dataset split.

Classes	Label	Training Samples (80%)	Validation Samples (10%)	Test Samples (10%)
3	Normal	576	72	72
	IR	576	72	72
	OR	576	72	72
	Total	1728	216	216

**Table 7:** Diagnosis accuracies.

Model	Training Accuracy	Validation Accuracy	Test Accuracy	Hardware	Training Time (s)
**VGGish Transfer Learning**	**100.00%**	**100.00%**	**99.07%**	**Intel® Core i7 − 10510U CPU @ 1.80 GHz**	**936**
VGGish from scratch	50.00%	33.33%	33.33%	${\mathrm{Intel}}^{®}\;\mathrm{Core}\;i7\;-$ 10510U CPU @ 1.80 GHz	1038
YAMNet [56]	100.00%	99.07%	91.20%	${\mathrm{Intel}}^{®}\;\mathrm{Core}\;i7\;-$ 10510U CPU @ 1.80 GHz	264
VGG16 [55]	53.12%	66.20%	69.44%	GPU NVIDIA^®^ T4	693

**Table 8:** Precision and recall of the diagnosis models.

Model	Label	Precision	Recall
	Normal	98.61%	98.61%
VGGish Transfer Learning	IR	100.00%	100.00%
	OR	98.61%	98.61%
	Normal	33.33%	100.00%
VGGish from scratch	IR	0.00%	0.00%
	OR	0.00%	0.00%
	Normal	100.00%	73.60%
YAMNet [56]	IR	100.00%	100.00%
	OR	79.10%	100.00%
	Normal	67.90%	79.20%
VGG16 [55]	IR	69.40%	59.70%
	OR	71.40%	69.40%

## Data Availability

The data are not publicly available due to the policy of the department.
